# Reconsidering the public health failings of the criminal justice system: a reflection on the case of Scott Ortiz

**DOI:** 10.1186/1477-7517-3-25

**Published:** 2006-08-15

**Authors:** Thomas Kerr

**Affiliations:** 1 British Columbia Centre for Excellence in HIV/AIDS, St. Paul's Hospital, 608-1081 Burrard Street, Vancouver BC V6Z 1Y6, Canada; 2 Department of Medicine, University of British Columbia, 3300-950 West 10th Avenue, Vancouver BC V5Z 4E3, Canada

## Abstract

Throughout most of the world, the primary response to the health and social impacts of illicit drug use has been to intensify the enforcement of drug laws. The consequences of this policy approach include an unprecedented growth in prison populations and increasing concerns regarding drug-related harms within prisons and without, including increased risk of HIV and hepatitis C (HCV) infection. This has led to calls from public health and prisoner advocacy groups to prison authorities to improve health services available in the community and those available to prisoners. While considerable progress has been made with respect to the growing implementation of HIV and HCV prevention measures within some nations' prisons, the case of Scott Ortiz illuminates a new set of challenges for prisoners and their advocates as judges often have a faulty understanding of public health arguments and data. In this case we see one such instance where a judge acts in ways not rooted in sound public health evidence or practice to produce a perverse outcome that violates both sound medical and judicial objectives.

## Background

Throughout most of the world, the primary response to the health and social impacts of illicit drug use has been to intensify the enforcement of drug laws in an effort to limit the supply and use of illicit drugs [1]. The consequences of this policy approach include an unprecedented growth in prison populations and increasing concerns regarding drug-related harms within prisons [2]. In recent years, incarceration has been associated with an array of harms, including increased risk of HIV and hepatitis C (HCV) infection that results from injecting that occurs in prisons in the absence of effective prevention measures such as syringe exchange programs [3]. This has led to calls from public health and prisoner advocacy groups to prison authorities to honor the 'principle of equivalence' which states that health services available in the community must also be made equally available to prisoners [3].

While considerable progress has been made with respect to the growing implementation of HIV and HCV prevention measures within prisons, the case of Scott Ortiz illuminates a new set of challenges for prisoners and their advocates. Mr. Ortiz is described as a former injection drug user who had been convicted of burglary. Upon conclusion of Mr. Ortiz's trial, the presiding judge imposed an extraordinary and lengthy sentence based on a public health argument that was not rooted in sound public health evidence or practice. In short, Mr. Ortiz was convicted as a means of reducing the likelihood that he might transmit his infectious diseases to others through illicit drug use. Aside from being tragic, this decision was also ironic given what is known about the high risk injecting environments within prisons. If Mr. Ortiz was in fact an active injector or a past injector who returned to injecting within prison, it is clear that greater individual and public health-related harm would result from incarcerating him. But, more importantly, the sentence given to Mr. Ortiz suggests that, even when there is no clear legal or public safety rationale for lengthy incarceration, former or current injection drug users may face significant discrimination and potential harm through sentencing erroneously designed to protect public health.

The use of sentencing of injection drug users to protect public health represents a rather disturbing development in the realm of drug policy and illustrates the extent to which dominant social narratives that portray drug users as reckless and lacking regard for the health of others have penetrated the judiciary. This is particularly disturbing given the power and independence afforded to the judicial system. Further, the case of Mr. Ortiz also demonstrates how the blurring of criminal justice and health systems responses to drug use seems to continuously present new harms, as custody and control repeatedly trump efforts to protect and promote individual health. Given the current dominance of enforcement and incarceration in drug policy, the case of Mr. Ortiz suggests new work for public health practitioners, prisoner advocates, and legal reformers, with ignorance and discrimination within the judiciary being the main target for action.

Correction is a public safety rather than a public health activity, and therefore the justice system and prison life itself are not organized in accordance with public health principles. Prevention and care of diseases does, in some instances, require the difficult task of reconciling or balancing a public health model of prevention, diagnosis, care, and treatment with the correctional requirements of custody and control [4]. However, such a balancing act in no way indicates a role for the judiciary in preventing infectious disease transmission by incarcerating those whom an individual judge deems to pose a risk as a result of their past or current illicit drug use. Let us only hope that the tragic story of Mr. Ortiz ignites new action that ultimately serves to prevent or at least limit the use of law and order as a tool of public health.
